# Religious Social Capital and Perceived Health among US Older Adults: A Path Analysis Model

**DOI:** 10.1093/geroni/igaf122.3130

**Published:** 2025-12-31

**Authors:** Seoyoon Woo, Jeonghwa Lee, Yeoun Soo Kim-Godwin, Jeeyae Choi

**Affiliations:** University of North Carolina Wilmington, Wilmington, North Carolina, United States; The University of North Carolina at Wilmington, Wilmington, North Carolina, United States; The University of North Carolina at Wilmington, Wilmington, North Carolina, United States; The University of North Carolina at Wilmington, Wilmington, North Carolina, United States

## Abstract

Rationale/Objectives--Perceived health and social connectedness are significant indicators of overall health status and well-being among older adults. This study aimed to examine the relationship between religious/spiritual belonging and perceived physical and mental health among older adults in the US. Methods--This secondary cross-sectional study included 2,163 adults aged 50–80(mean=64.5) from the National Poll on Healthy Aging(Wave11), a nationally representative U.S. health survey. Independent variables included religious/spiritual belonging, chronic health conditions, healthy eating habits, BMI, and age, while dependent variables were perceived physical and mental health. Mediators examined were confidence in helping others, feelings of isolation, lack of companionship, and frequency of social contact. Path analyses were conducted using the lavaan package in R. Results--All subjects have some form of religion: 56.4% feel they are part of a community that shares their beliefs. The path model for mediation demonstrated a good fit to both perceived physical health (Χ^2(17)=85.136, p < 0.001; RMSEA = 0.065, 90% CI[0.051-0.079]; CFI = 0.950; SRMR = 0.050; R^2 = 0.295) and perceived mental health (Χ^2(13) = 75.253, p < 0.001; RMSEA=0.071, 90% CI[0.056-0.087]; CFI = 0.946; SRMR = 0.055; R^2 = 0.287). Having chronic health conditions (β = 0.19, p < 0.001), healthy eating habits (β=-0.24, p< 0.001), and BMI (β = 0.02, p < 0.001) have a direct effect on perceived physical health. Religious/spiritual belonging (β = 0.16,p=0.004), healthy eating habits (β=-0.15,p=0.004), and age (β=-0.01,p=0.011) have a direct effect on perceived mental health. Religious/spiritual belonging has an indirect effect on perceived physical and mental health, being mediated by confidence in helping others, feelings of isolation, lack of companionship, and frequency of social contact. Conclusion--Findings enhance the understanding of religious social capital’s impact on perceived physical and mental health through mediating factors.

